# Supplementary material for: What factors contribute to the need for physical restraint in institutionalized residents in Taiwan?
Source: PLoS One. 2022 Nov 17;17(11):e0276058. doi: 10.1371/journal.pone.0276058 (PMC9671315; doi:10.1371/journal.pone.0276058)
Supplement: Attachment — Submitted filename: Response to Reviewers 0710 (1).docx [file pone.0276058.s001.docx]

Response to Reviewers

Reviewers' comments:

Reviewer #1: The authors present an epidemiological study examining the factors that contribute to the need for physical restraint in institutionalized residents in Taiwan. The study is interesting but can be sharpened further. Please see my comments below:

**1. A key concern is the reporting of the study. It will be helpful if the authors use a standardized reporting guideline to report the study. As it stands now, the study details are mixed. For instance, under study subjects, the authors report information regarding data collection. Please rectify these.**
Answer:

The inappropriate content was removed. And the structure of paragraph was adjusted to meet the standardized reporting guideline. In addition, during the revision process, we found some minor data errors in the people number, so we also arranged correction. Sorry for the extra-burden on proofreading.

**2. It remains unclear if the outcome measures used are standardized. If they are, it will be helpful to provide more details regarding the measures such as the Cronbach's alpha.**
Answer:

The outcome in this study (having physical restraint) was measured by a single question: were you having physical restraint at the institution; if the answer is yes, the frequency of physical restraint was also recorded (please see the manuscript line 131-135) (revise with tracked change: line 132-138). The content validity of this question and the entire questionnaire used in this study has been approved by an expertise committee before the survey. Because the investigated outcome (physical restraint) was measured by a single question, we cannot calculate related statistics such as Cronbach's alpha.

**3. The exact statistical tests performed should be mentioned.**

Answer:

Please see the attached revised manuscript (line 168-176)(revise with tracked change: line 171-181) for the detailed description of statistical tests used in this study.

**Reviewer #2: The reason to conduct the study is not strong enough. What the authors found in the study probably can be predicted and had been studied previously in other countries. The authors need to sharpen why they need to conduct the study, particularly in the context of improving the situation. physical restraint should be also viewed not only from physical or psyschological aspects, but also from socio-spiritual aspects. It needs to be discussed clearly in the background, results, and discussion.**

Answer:

Thank you for the comment. We want to survey the physical restraint rate in institutions due to no common regulation in Taiwan. In the view of health care worker, we thought the rate may be higher than we think, and the result might help us to figure ways to improve the rate. We added socio-spiritual aspects on physical restraint in the text.
