# Supplementary material for: What factors contribute to the need for physical restraint in institutionalized residents in Taiwan?
Source: PLoS One. 2022 Nov 17;17(11):e0276058. doi: 10.1371/journal.pone.0276058 (PMC9671315; doi:10.1371/journal.pone.0276058)
Supplement: Attachment — Submitted filename: Response to Reviewers 0920.docx [file pone.0276058.s002.docx]

**Response to Reviewers**

**Reviewer #1: Most of the comments previously raised have been addressed. As a minor comment, please report the study using an appropriate reporting guideline such as the STROBE.**

**Answer:** Thank you for your suggestion. We re-examined the content of this article according to the STROBE guidelines, the results are attached below. We also made several improvements of the content.

STROBE Statement—Checklist of items that should be included in reports of ***cross-sectional studies***

|  | Item No | Recommendation | Article Page |
| --- | --- | --- | --- |
| **Title and abstract** | 1 | (*a*) Indicate the study’s design with a commonly used term in the title or the abstract | 1,3 |
|  |  | (*b*) Provide in the abstract an informative and balanced summary of what was done and what was found | 3-4 |
| Introduction | | |  |
| Background/rationale | 2 | Explain the scientific background and rationale for the investigation being reported | 5-7 |
| Objectives | 3 | State specific objectives, including any prespecified hypotheses | 7 |
| Methods | | |  |
| Study design | 4 | Present key elements of the study design early in the paper | 7-8 |
| Setting | 5 | Describe the setting, locations, and relevant dates, including periods of recruitment, exposure, follow-up, and data collection | 7 |
| Participants | 6 | (*a*) Give the eligibility criteria, and the sources and methods of selection of participants | 7 |
| Variables | 7 | Clearly define all outcomes, exposures, predictors, potential confounders, and effect modifiers. Give diagnostic criteria, if applicable | 8-10 |
| Data sources/ measurement | 8* | For each variable of interest, give sources of data and details of methods of assessment (measurement). Describe comparability of assessment methods if there is more than one group | 8-10/ 11 |
| Bias | 9 | Describe any efforts to address potential sources of bias | 22 |
| Study size | 10 | Explain how the study size was arrived at | Not applicable |
| Quantitative variables | 11 | Explain how quantitative variables were handled in the analyses. If applicable, describe which groupings were chosen and why | 11 |
| Statistical methods | 12 | (*a*) Describe all statistical methods, including those used to control for confounding | 11 |
|  |  | (*b*) Describe any methods used to examine subgroups and interactions | Not applicable |
|  |  | (*c*) Explain how missing data were addressed | missing data was not included in the analysis |
|  |  | (*d*) If applicable, describe analytical methods taking account of sampling strategy | Not applicable |
|  |  | (*e*) Describe any sensitivity analyses | Not applicable |
| Results | | |  |
| Participants | 13* | (a) Report numbers of individuals at each stage of study—eg numbers potentially eligible, examined for eligibility, confirmed eligible, included in the study, completing follow-up, and analysed | 11 |
|  |  | (b) Give reasons for non-participation at each stage | 797 cases in our study failed to complete the survey, and 1 case did not fulfill the medical history item |
|  |  | (c) Consider use of a flow diagram | We did not use due to abundant tables |
| Descriptive data | 14* | (a) Give characteristics of study participants (e.g. demographic, clinical, social) and information on exposures and potential confounders | 12 |
|  |  | (b) Indicate number of participants with missing data for each variable of interest | Not applicable |
| Outcome data | 15* | Report numbers of outcome events or summary measures | 12-19 |
| Main results | 16 | (*a*) Give unadjusted estimates and, if applicable, confounder-adjusted estimates and their precision (e.g. 95% confidence interval). Make clear which confounders were adjusted for and why they were included | 12-19 |
|  |  | (*b*) Report category boundaries when continuous variables were categorized | Not applicable |
|  |  | (*c*) If relevant, consider translating estimates of relative risk into absolute risk for a meaningful time period | Not applicable |
| Other analyses | 17 | Report other analyses done—eg analyses of subgroups and interactions, and sensitivity analyses | Not applicable |
| Discussion | | |  |
| Key results | 18 | Summarise key results with reference to study objectives | 20-21 |
| Limitations | 19 | Discuss limitations of the study, taking into account sources of potential bias or imprecision. Discuss both direction and magnitude of any potential bias | 25-26 |
| Interpretation | 20 | Give a cautious overall interpretation of results considering objectives, limitations, multiplicity of analyses, results from similar studies, and other relevant evidence | 26 |
| Generalisability | 21 | Discuss the generalisability (external validity) of the study results | Not applicable |
| Other information | | |  |
| Funding | 22 | Give the source of funding and the role of the funders for the present study and, if applicable, for the original study on which the present article is based | 26-27 |

**Reviewer #2: Since elderly has many changes in the body, psycho-socio-spiritual aspects of elderly should be considered in studying treatment or intervention for them.**

**Answer:** With increased age, elderly people are prone to have a cognitive decline, dementia, multiple chronic disease, depression and delirium. Their resistance to external pressure are weaker than at younger age (Garrido P, De Blas M, Giné E, Santos Á, Mora F. Aging impairs the control of prefrontal cortex on the release of corticosterone in response to stress and on memory consolidation. Neurobiol Aging. 2012 Apr;33(4):827.e1-9. doi: 10.1016/j.neurobiolaging.2011.06.011. Epub 2011 Jul 27. PMID: 21794953) The use of physical restraints could be a stress, even resulting in fear and angryness of elderly people. The ego-integrity, good social support, family relationship, regular exercise habit and religions are ways to help people cope with stress and low mood. However, these helpful conditions are usually lacking in institutionalized elderly people. A review in 2014 revealed that patients had a tendency to develop deteriorated ADL, cognitive impairment and more agitation after being restrained (Hofmann H, Hahn S. Characteristics of nursing home residents and physical restraint: a systematic literature review. J Clin Nurs. 2014 Nov;23(21-22):3012-24. doi: 10.1111/jocn.12384. Epub 2013 Oct 11. PMID: 24125061) It is also found untreated psychotrauma in elderly could complicate with sleep disturbance, depression, suicide, and late-life delusional disorder (Flannery RB Jr. Restraint procedures and dementia sufferers with psychological trauma. Am J Alzheimers Dis Other Demen. 2003 Jul-Aug;18(4):227-30. doi: 10.1177/153331750301800408. PMID: 12955787)
